# Relapsed Primary Central Nervous System Lymphoma: Current Advances

**DOI:** 10.3389/fonc.2021.649789

**Published:** 2021-04-29

**Authors:** Kaiyan Tao, Xuefeng Wang, Xin Tian

**Affiliations:** Department of Neurology, The First Affiliated Hospital of Chongqing Medical University, Chongqing Key Laboratory of Neurology, Chongqing, China

**Keywords:** primary central nervous system lymphoma, relapse, mechanism, clinical feature, treatment, prognosis

## Abstract

Primary central nervous system lymphoma is an invasive malignant lymphoma confined to the central nervous system. Although patients undergoing first-line treatment can achieve complete response, most of them still relapse within two years. Relapsed lymphoma is derived from occult lymphoma cells, and B cell receptor pathway activation and immune escape are the key mechanisms for the pathogenesis of PCNSL. Most relapses are in the central nervous system, a small number of relapses are isolated systemic relapses, and clinical symptoms occur early and vary. Current treatments for relapse include high-dose methotrexate rechallenge and other regimens of chemotherapy, whole-brain radiation therapy, hematopoietic stem-cell transplantation, targeted therapy and immunotherapy, which have become promising treatments. The overall prognosis of relapsed PCNSL is very poor, although it is affected by many factors. This article summarizes the mechanisms, related factors, clinical features, follow-up, treatment and prognosis of relapsed primary central nervous system lymphoma.

## Background

Primary central nervous system lymphoma (PCNSL) is a highly malignant non-Hodgkin’s lymphoma (NHL) that originates in the central nervous system (CNS) and eyes. Diffuse large B-cell lymphoma (DLBCL) is the most common subtype of PCNSL, followed by lymphoma of T cell or natural killer cell origin. PCNSL accounts for 2-3% of NHL and 4% of CNS malignancies (1–3). Despite significant improvements in the management of PCNSL, up to 60% of patients eventually relapse, and there is currently no accepted standard salvage regimen, additionally, the rate of remission is low, while prognosis after relapse remains poor (4–6). This article summarizes the mechanisms, clinical features, follow-up, treatment and prognosis of relapsed PCNSL, aiming to provide evidence for further understanding and designing better treatment strategies.

## Historical Evolution

**Figure 1:**
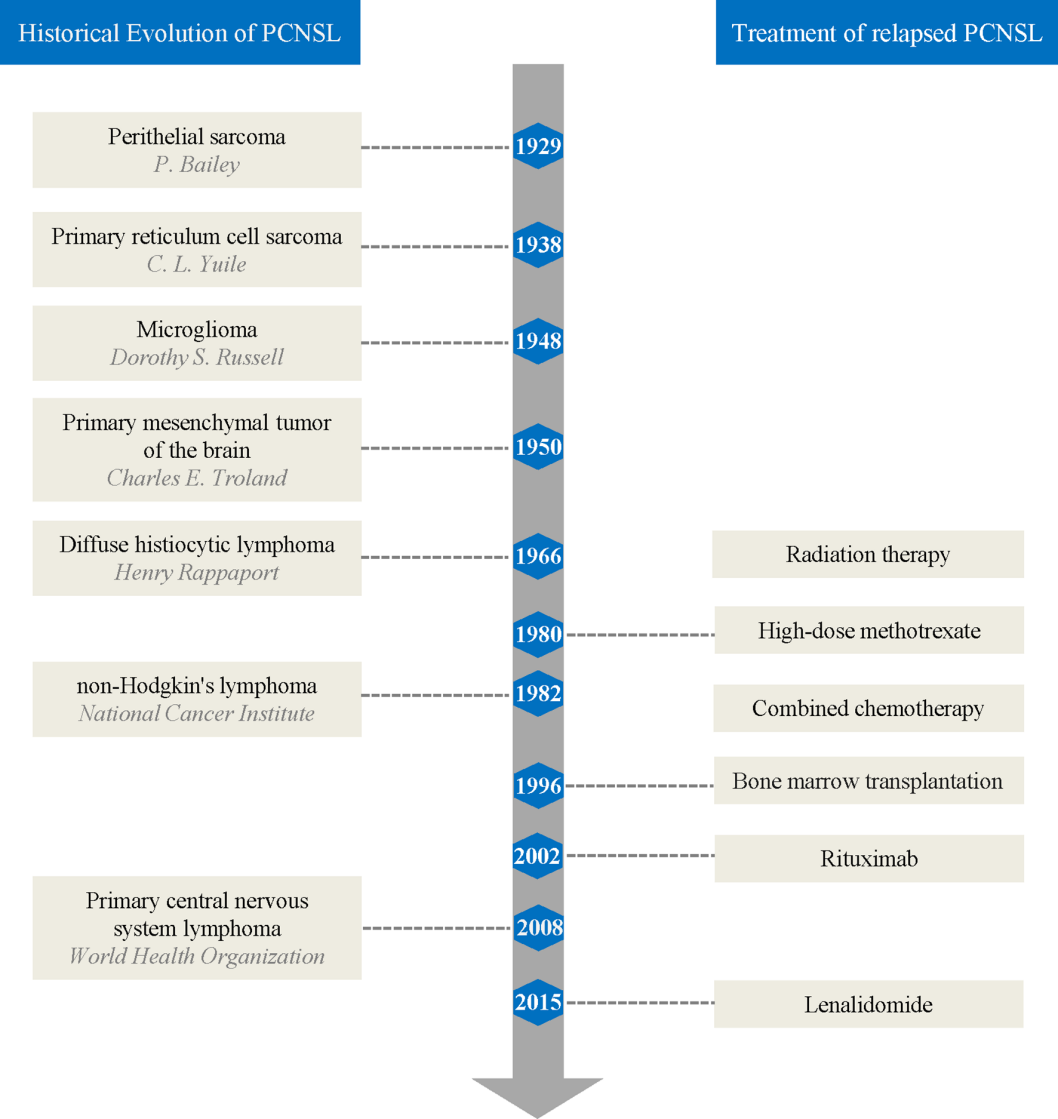
Brief summary of primary central nervous system lymphoma history and treatment of relapses.

The historical evolution of PCNSL nomenclature reflects the evolving understanding of the pathology of PCNSL among researchers (**Figure 1**). In 1929, Bailey (7) first described PCNSL and referred to it as “perithelial sarcoma”. In 1938, Yuile (8) first reported the complete autopsy results of a PCNSL case in which the tumor cells were identical to reticulum sarcoma in other parts of the body and two cases of perithelial sarcoma published by Bailey, known as brain “primary reticulum cell sarcoma”. In 1948, Russell and colleagues (9) termed this cancer “microglioma” based on research suggesting that silver carbonate had affinity for microglia rather than for primitive reticulocytes. In 1950, Troland and colleagues (10) observed that the reticulum was not a tumor component in their cases and proposed the term “primary mesenchymal tumor of the brain” based on the similarity in morphology and behavior of tumor cells to mesenchymal tissues. By 1966, Rappaport (11) proposed the first clinically relevant classification of lymphoma based on cell morphology, and PCNSL was called “diffuse histiocytic lymphoma”. In 1982, the National Cancer Institute defined NHL, replacing Rappaport’s classification (12). In 1994, the International Lymphoma Study Group proposed the Revised European-American Classification of Lymphoid Neoplasms (13) and contributed to the 2001 edition of the World Health Organization (WHO) lymphoma classification. Finally, the 2008 edition of the WHO classification of tumors of hematopoietic and lymphoid tissues used PCNSL as a distinct entity (14).

## Mechanism

The mechanism of relapsed PCNSL is unclear, and one potential mechanism is occult PCNSL cell dissemination beyond the blood-brain barrier (BBB). Muldoon et al. (15) found that rituximab (RTX) reduced the tumor volume at the inoculation site and prolonged the survival of rats, but the tumor still spread to the subdural space, the contralateral ventricle, the base of the brain and the cortex. Doolittle et al. (16) also observed that in patients with PCNSL receiving 90Y-zevalin, multiple tumor relapses distant from the initial site took place with no active tumors at the initial site, and tumor relapse might be caused by poor penetrability of 90Y-zevalin through the intact BBB. Genomic analysis not only suggests a molecular mechanism of relapse but also supports the concept of both late relapses and isolated systemic relapses being derived from occult lymphoma cells, which include common precursor cells and clonality. Nayak et al. (17) examined clonal rearrangements in the histological specimens of primary and relapsed tumors of a patient with PCNSL who relapsed after 13.8 years and found that both tumors had the same clonality. The study by de Jong et al. (18) also supported the same clonal relationship between primary and relapsed tumors. Notably, extra-CNS relapses of PCNSL, especially “early systemic relapses”, may be derived from occult systemic lymphoma at the time of initial diagnosis, rather than being a true relapse, and studies supported the presence of occult systemic lesions at the time of initial diagnosis in up to 12.5% of patients with PCNSL (4, 19, 20). In addition, extra-CNS subclinical lesions of PCNSL that could not be eradicated by first-line treatment are easy to confuse with relapses (21), especially “very early relapses”.

Both primary and recurrent tumors originated from common precursor B cells, but the relapsed tumor was not directly derived from the primary tumor. Clonal evolution rather than subclonal selection was the basis of relapses in this condition, namely, lymphoma cell clonality was not completely consistent with that of primary lymphoma cells after different accumulations and evolutions during mutations. Pels et al. (22) compared the genetic test results of a primary and relapsed brain tumor in a 71-year-old PCNSL patient and showed that both tumors had the same Ig gene segments. However, although both tumors shared somatic mutations in 22 rearranged Ig genes, they also carried distinct mutations, and sequences in the relapsed tumor were not present in the primary tumor. A study by Garcia-Reyero et al. (23) compared a primary tumor in the CNS with an extra-CNS recurrent tumor. Although these tumors had the same morphology, phenotype, cytogenetic characteristics, clonal relationship and MYD88 L265P mutations, the relapsed tumor had a distinct CD79B Y196S mutation, suggesting that both tumors might have evolved from a common progenitor clone with MYD88 L265P mutations. Hattori et al. (24) further elucidated the characteristics of somatic mutations and detected SOCS1 (primary skin DLBCL-associated) in a recurrent tumor in the subcutaneous tissue after targeted deep sequencing of primary intra-CNS tumors and isolated extra-CNS recurrent tumors from 5 PCNSL patients, suggesting that somatic mutations may be biased toward the lymphoma site.

Regardless of clonal selection or clonal evolution, recurrent lymphoma cells have undergone genetic and epigenetic changes in which activation of the B cell receptor (BCR) and Toll-like receptor (TLR) pathways, Janus kinase/signal transducers and activators of transcription (JAK/STAT), and immune escape are the key mechanisms of PCNSL; these alterations are the targets of new therapies. Next-generation sequencing analysis showed that more than 80% of PCNSL patients had mutations in 8 genes (CTNNB1, PIK3CA, PTEN, ATM, KRAS, PTPN11, TP53, and JAK3), and SMO gene mutations were associated with early disease recurrence (25). The BCR and TLR signaling pathways are often activated by MYD88 and CD79B gene mutations. The L265P mutation of MYD88 occurs in 67-72% of patients, and the CD79b mutation is present in 61% of patients (26). Activation of the BCR pathway promotes the subsequent activation of the phosphoinositide 3-kinase (PI3K)/AKT/mammalian target of rapamycin (mTOR) pathway (27), and the activation of both the BCR and TLR pathways can also lead to enhanced nuclear factor kappa-b (NF-κB) activity. In addition, other genes in the NF-κB pathway are also highly enriched in aberrant somatic hypermutation targets and candidate driver factors (such as PIM1, BTG2, CD44, XBP1 and NFKBIE) of PCNSL (28). Abnormal activation of the JAK/STAT pathway is also involved in the pathogenesis of PCNSL. STAT3 phosphorylation (STAT3 activation) and increased expression of interleukin-10 (IL-10) were detected in 53.1% and 78.1% of PCNSL tumor samples, respectively. IL-10 expression is related to STAT3 phosphorylation, and the latter is significantly associated with poor prognosis of PCNSL (29, 30). Epigenetic silencing of important functional genes is also important in the development of PCNSL. PCNSL patients have at least 2 methylated genes; 96% of tumors have DAPK, p16 (INK) (4a) or MGMT methylation; and relapsed patients with MGMT methylation show a response to temozolomide (31, 32). Immune escape may be important in PCNSL pathophysiology. Recent studies have shown that 9p24.1 copy number alterations frequently occur, and the associated programmed cell death protein 1 (PD-1) ligands 1 (PD-L1) and PD-L2 are increased in PCNSL. The chromosomal rearrangement of PD-L1 and PD-L2 and the selective overexpression of the corresponding receptors have been identified (33).

## Relapse Rate and Relapse-Related Factors

### Relapse Rate and Time

PCNSL is an invasive NHL, and although some patients can achieve complete response (CR) after initial first-line treatment, 36-66.6% of patients still relapse (4, 17, 34, 35). Jahnke et al. (35) evaluated the clinical characteristics of 143 patients with PCNSL who achieved CR after initial treatment, and 36% of them relapsed after a median follow-up of 22.5 months. Yamanaka et al. (4) conducted an average of 14 years of follow-up in 114 patients with PCNSL and found that 66.6% of the patients relapsed after a median duration of 14 months after PCNSL diagnosis.

Most of the first relapses occur within two years after the initial diagnosis of PCNSL (median 10-18 months), with a small number of late relapses (≥5 years) (17, 35). A study by Yamanaka et al. (4) suggested that 63.3% of 60 patients with PCNSL relapsed within two years, 11.6% relapsed within 5-10 years, and 3.3% relapsed after 10 years; the median time to late relapse was 7.4-7.5 years (17).

### Relapse-Related Factors

There are many related factors for recurrence, including age, intraocular involvement, and initial treatment. A study by Langner-Lemercier et al. (6) suggested that 77.7% of patients with relapsed/progressive PCNSL were over 60 years old, patients with late relapses were younger—60% were less than 50 years old, and the median age at the time of initial diagnosis was 47 years (17).

Patients with intraocular lymphoma (IOL) may be prone to relapse. Kreher et al. (36) compared 19 IOL+ patients with 278 IOL- patients with PCNSL and found that there was a significant difference in the median progression-free survival (mPFS) between these two groups (3.5 months and 8.3 months, respectively). Multivariate analysis indicated that patients with IOL had significantly worse progression-free survival than patients without intraocular involvement. A prospective cohort study of 103 patients with PCNSL by Zhuang (37) indicated that the relapse rates of patients with and without intraocular involvement were 71.4% and 46.3%, respectively, suggesting that patients with IOL were more likely to relapse.

For patients with isolated primary vitreoretinal lymphoma (PVRL), compared with local therapy alone, combination therapy could significantly delay lymphoma relapse. Klimova et al. (38) retrospectively compared local therapy alone with combination therapy (local and systemic) among 10 patients with PVRL and found that initial combination therapy in PVRL patients significantly prolonged the time to first relapse.

Although most patients experience remission after initial treatment, increased relapse can be observed with prolonged follow-up, and consolidation therapy maintains the state after induction therapy; thus, initial consolidation therapy may play an important role in relapsed PCNSL (39, 40). Chanswangphuwana et al. (40) retrospectively analyzed the effect of whole-brain radiation therapy (WBRT) as a consolidation therapy in patients with PCNSL relapse, and some of the 37 patients with newly diagnosed PCNSL underwent WBRT after initial remission. The results showed that among the 22 patients with CR, PFS was significantly lower in patients without WBRT than in patients with WBRT, and the 3-year PFS rates were 35% and 78.75%, respectively.

## Clinical Features

### Relapse Pattern

Of the first relapses, 84.6-91.3% of PCNSL are only sites in the CNS (including the eye), 8.7-16% are isolated systemic relapses, most of which are extranodal, and very few are CNS and systemic relapses (19, 35, 41). Jahnke et al. (35) evaluated the clinical features of 52 patients with relapsed PCNSL and found that 44 patients had CNS relapses only, 6 patients had systemic relapses only, and 1 patient had both CNS and testicular relapses. Most of the recurrences were in the brain parenchyma, and a few were in the meninges, spinal cord parenchyma and eyes (34). Provencher et al. (19) examined the data from 115 patients with relapsed PCNSL and found that 92 patients had recurrence in the brain (6 leptomeningeal involvement and 1 ocular involvement), 1 patient experienced relapse in 3 regions, namely, the brain, eye and leptomeninges, 3 and 7 patients had isolated ocular and meningeal relapses, respectively, 2 patients had single leptomeningeal relapse, and 10 patients had EC relapses. A retrospective study by Mao et al. (42) supported the above conclusions.

Most intracranial relapses are located at a distance from the initial tumor site. The pattern of distant relapse is unique to the natural history of PCNSL, and recognizing patterns of relapse is key for early detection. Schulte-Altedorneburg et al. (43) first evaluated the relapse pattern of 16 PCNSL patients and found that distant, cortex and subcortex were the most common sites of recurrence in 12 patients who relapsed, while ventricular and subependymal MRI contrast enhancement patterns were most common non-parenchymal patterns. The retrospective study of Ambady et al. (41) also supports this result; the imaging of 37 patients with recurrent PCNSL showed that 81% of patients relapsed distant from the initial tumor site; 50% in different brain lobes; 30% in the eyes, different parts of the same lobe, corpus callosum and pia mater; 20% in extra-CNS sites; and the remaining 19% near the primary site (within 2 cm of the T2 high density at the initial diagnosis), especially when the initial lesion involved the corpus callosum, posterior fossa, subependymoma, or meningitis. A prospective study by Tabouret et al. (44) observed that >50% of PCNSL patients relapsed at a certain distance from the initial tumor site, with 46% of the brain recurrence located at the initial enhancement site, 40% far away from the initial site, and 14% both were both. The number of lesions, topography, and T1 volume were not different from the primary tumor. The CNS pattern in late relapses is similar to that in early relapses—mainly in the brain—but most of the sites are distant from the initial ones (17). These studies further supported that seeding from occult lesions or other extra CNS sites is the mechanism of relapse, suggesting the importance of identifying treatment strategies that cross the blood-brain barrier to reach PCNSL invasion sites.

Sites of isolated systemic relapses include lymph nodes (lung, ventral, paratracheal, retroperitoneal, and cervical), the musculoskeletal system, testis, bone marrow, kidney and adrenal structures, extranodal lymphoid tissue, liver, small intestine, and abdominal wall, but lymph nodes, the musculoskeletal system, testis and bone marrow are the most common (19, 35, 45). Isolated systemic relapses can also occur in rare sites. Chuang et al. (46) reported a case of an 82-year-old PCNSL patient with two consecutive relapses in the dermis and subcutaneous tissue without local failure or other systemic involvement, and three tumors showed the same clonal origin. Ahmed et al. (47) reported a case of relapse with a 1.5-cm isolated subcutaneous nodule at the original lumbar puncture site after 2.5 years of initial remission in a 63-year-old male PCNSL patient. Partial late relapses are systematically involved, and the sites of recurrence include the nasal cavity, mouth, throat, mediastinum, gastrointestinal tract, breast, lung, spleen and pelvis (4).

### Clinical Manifestation

Most patients have clinical symptoms at relapse, and cognitive impairment and paralysis are the most common (48). Langner-Lemercier et al. (6) showed that 74.5% of patients had symptoms during relapse/progression, and symptoms based on the frequency of occurrence were gait disorder (59.5%), cognitive impairment (55.5%), sensorimotor disorder (47.1%), balance disorder (45.9%), aphasia (16.2%), increased intracranial pressure (15.7%), and epilepsy (4.5%). Fossard et al. (49) also showed that 80.3% of relapses were symptomatic, including cognitive impairment (43%), motor impairment (14%), increased intracranial pressure (11%), altered performance status (11%), visual impairment (9%), and epilepsy (5%). However, the clinical features of age, relapse site, meningeal involvement, cerebrospinal fluid (CSF) protein level, and lactate dehydrogenase level did not differ between patients with and without symptoms.

In addition, neuronal lymphoma (NL) (in which lymphoma cells infiltrate the peripheral nervous system) can be a rare form of PCNSL relapse. The International Primary CNS Lymphoma Collaborative Group retrospectively analyzed 50 patients with NL, 9 of whom showed relapsed/progressive PCNSL (50). Le Guennec et al. (51) reported a case of a 60-year-old female with PCNSL with CR for 3 years who was admitted to the hospital with severe pain in the left leg due to sagging of the foot. Electroneuromyography showed left S1 radiculopathy, and MRI suggested thickening of the left sciatic nerve root and strengthening of the sciatic nerve. Biopsy confirmed invasive large CD20^+^ B cells in the intimal tissue and PCNSL relapse.

A common pattern of PCNSL relapse is CSF dissemination. Patients may present with lymphomatous meningitis (LM), although isolated leptomeningeal relapse is also not common. Relapse at both the brain parenchyma and meninges can be as high as 40% (52). Chamberlain et al. (53) retrospectively evaluated 14 patients (median age of 56 years) with recurrent PCNSL presenting with LM or brain parenchyma tumors combined with CSF-disseminated tumors. The clinical manifestations included altered mental status (71%), headache (50%), cranial nerve defects (36%), gait ataxia (29%), visual field defects (29%), seizures (14%) or hemiplegia (14%). The most common manifestations of LM involving the cerebral hemisphere are headache and altered mental status. When the cranial nerve is involved, the abductor nerve is the most affected. When the spinal cord is involved, the manifestations include weakness (lower limbs more than upper limbs), skin or segmental sensation loss, and neck or root pain (52).

## Follow-Up After Initial Treatment

Although the first relapse of PCNSL seldom occurs after 5 years, the American Society of Clinical Oncology recommends that follow-up should be performed for 10 years. The most basic examinations at follow-up should include a medical history, physical examination (including Mini-Mental State Examination), and brain-enhanced MRI scan (54). According to the National Comprehensive Cancer Network (NCCN) guidelines version 3.2020, follow-up after initial treatment should be performed using brain MRI every 3 months until 2 years, every six months until 5 years, and then annually indefinitely. For patients with spinal cord involvement, spinal imaging and CSF sampling should be simultaneously performed, while ophthalmic follow-up is also required for patients with ocular involvement (55). Most asymptomatic relapses are found with neuroimaging, and a small number are found with ophthalmologic examination. Very few relapses are found with CSF analysis, but when relapses are transmitted through the CSF, the CSF detection rate is increased (6, 53), and cerebrospinal fluid cytology is the gold standard for the detection of malignant leptomeningeal invasion (56). As systemic relapse is likely rare, studies suggest that periodic systemic assessment of extracranial sites may not always be required (34).

Small PCNSL lesions can cause serious symptoms, making it difficult to detect preclinical relapses by routine brain MRI, so careful assessment of symptoms by clinicians is far more important than relying on regular imaging examination (48, 49). Fossard et al. (49) followed 61 patients with PCNSL who achieved CR after initial treatment. Among them, 49 patients first had symptoms at relapse and 12 relapses were detected by routine MRI before symptoms appeared; moreover, only these 12 cases were detected from the total 819 brain MRI studies in the first 5 years. Mylam et al. (48) also evaluated the value of MRI to detect relapsed PCNSL. In their study, of 32 patients, 30 patients underwent MRI due to new clinical symptoms, and only 1 relapse was detected with routine MRI studies, indicating that 1 out of 189 MRI studies showed preclinical relapse. Since most patients relapse within 2 years and symptoms appear early, the utility of routine MRI studies after the first 2 years of follow-up as advice in the guidelines is very limited. 18F-Fluorodeoxyglucose (FDG) positron emission tomography (PET) may be more sensitive than traditional imaging examination and systemic recurrence may be easier found by PET (57, 58), but there is still a lack of original research to prove its role in follow-up. A recent study showed that the diagnosis rate of systemic relapses by FDG PET was low, with a specific false positive rate, suggesting that there was no more benefit from FDG-PET compared to traditional imaging examinations (59).

For atypical MRI findings or for new brain lesions that appear early after the initial treatment, re-biopsy is recommended, especially when intensive salvage treatment is planned (60). Clonality analysis is reliable and can provide information on the source and mechanism of relapse at the same time (17, 22). Accessible CSF analysis can help confirm the diagnosis except for the risk of herniation caused by intracranial masses. Due to the low diagnosis rate of CSF cytomorphology, flow cytometry and clonality analysis, in recent years, research has focused on finding relapse predictive factors in CSF examinations for relapse early detection (41). The sensitivity of detecting IL-10 in the CSF of relapsed patients is high (78.95%), and the increase of IL-10 concentrations in the CSF is related to PCNSL relapse (61). For example, IL-10 expression became positive and increased in 62.5% of relapsed patients, earlier than the MRI changes (62). The following factors involved in the regulation of B cell homeostasis can reflect the progression of the disease, including the soluble: transmembrane activator CAML interactor (TACI) and that B cell maturation antigen (BCMA) from the tumor necrosis factor family in CSF significantly increased in relapsed patients compared to remission patients (63). A single-center prospective study by Mulazzani et al. (64) suggested that a proliferation-inducing ligand (APRIL) in the CSF increased in 100% of patients who relapsed after initial CR, with an average increase of 351%; thus, APRIL might be a predictive marker of relapse. Although these predictors are not yet definitive evidence of relapse, this finding provides a new perspective for PCNSL follow-up.

## Treatment

Radiation therapy was initially the only way to treat relapsed lymphoma in the brain. However, since Ervin et al. (65) first reported that a patient with relapsed PCNSL achieved CR after high-dose methotrexate (HD-MTX) rechallenge in 1980, methotrexate (MTX) alone or in combination with other chemotherapeutics became the main treatment cornerstone of relapsed PCNSL (66), followed by autologous bone marrow transplantation (67). In 2002, Pels et al. (68) reported on a patient with refractory PCNSL who was successfully treated with rituximab (RTX) both intravenously and intraventricularly. Since then, molecular-targeted drug-related studies have gradually increased in number, providing more treatment options for relapsed PCNSL (**Figure 1**).

### Guidelines

The guidelines of the European Association for Neuro-Oncology in 2015 recommend that salvage therapy for patients with relapsed PCNSL depends on age, performance status, relapse sites in the CNS, previous treatment, and duration of the last remission (69). The NCCN guidelines version 3.2020 recommend adjusting treatment strategies based on the patient’s initial treatment (HD-MTX/WBRT/high-dose chemotherapy followed by autologous stem cell transplantation (HDC-ASCT)) and response duration (12 months) and also recommend using lenalidomide, ibrutinib or lenalidomide combined with rituximab (55). According to the guidelines, first, patients should participate in clinical trials as much as possible. Second, patients who are qualified for intensive treatment should choose HD-MTX rechallenge and other chemotherapy regimens based on their initial response to HD-MTX. Finally, consolidation of ASCT or palliative treatment (including WBRT) should be chosen according to the response of intensive treatment (55, 60).

### Chemotherapy

#### Methotrexate-Based Chemotherapy

If the patient has achieved a response after initial methotrexate (MTX) treatment, especially lasting for over 12 months, MTX rechallenge is a safe and effective strategy for relapse (55). The overall response rate (ORR) of the first MTX rechallenge is 85-91%, the median overall survival (mOS) is 41-62 months, and the need for more toxic salvage therapy can also be delayed (70, 71). In a retrospective study by Plotkin et al. (70), 22 patients with relapsed PCNSL who achieved a response with HD-MTX initial treatment received HD-MTX (≥3 g/m^2^) rechallenge as a single regimen for two salvage therapies. The ORR for the first rechallenge was 91%, and that for the second rechallenge was 100%; moreover, the mOS after the first relapse was 61.9 months. A study by Pentsova et al. (71) also supported MTX-based salvage therapy for relapsed PCNSL; the ORR was 85%, the 1-year OS was 79%, and the mOS was 41 months.

#### Active Drugs Known to Cross the Blood-Brain Barrier

If patients have achieved a response in less than 12 months after HD-MTX treatment or are not suitable for WBRT, single or combination chemotherapeutics can be a second-line therapy (55, 69). PCV (procarbazine, lomustine, and vincristine) chemotherapy is used as a second-line treatment for relapsed PCNSL, and its mechanism is different from that of MTX; the ORR of PCV is 50-86% and the mOS is 8-16 months (72, 73). Procarbazine and lomustine have cumulative bone marrow toxicity, but temozolomide does not. Thus, temozolomide has replaced PCV chemotherapy for many indications and is suitable for patients with renal insufficiency who cannot be treated with HD-MTX as well as elderly patients. The reported mOS for temozolomide with or without RTX salvage treatment is 3.5-14 months (72, 74, 75). However, the efficacy of temozolomide combined with rituximab still needs further verification (76). High-dose cytarabine alone has limited activity and significant toxicity in relapsed PCNSL (77), but it is often combined with other chemotherapeutics due to its good CNS penetration and lack of cross-resistance with MTX (78). Chemotherapy based on ifosfamide is also a feasible and effective option, with a response rate of 41-70%, most of which are durable responses (6, 79, 80).

### Radiation Therapy

WBRT-naive patients who are not suitable for HDC-ASCT retreatment and some palliative treatment for first time WBRT should be considered for WBRT either after salvage chemotherapy or alone (60). Patients with recurrent PCNSL who have not previously received WBRT are sensitive to WBRT, and its efficacy may be comparable to many salvage chemotherapy regimens, but the significant delayed neurotoxicity caused by WBRT in patients over 60 years old should be noticed (81, 82). Hottinger et al. (82) retrospectively analyzed the outcome of salvage WBRT in 48 progressive/relapsed PCNSL patients, and the results showed that 58% of patients achieved radiological CR, 21% achieved partial response (PR), 6% had stable disease, the median survival time was 16 months, and 22% of patients had delayed neurotoxicity, especially in patients over 60 years old and patients who had received MTX treatment for less than 6 months, showing cognitive symptoms with white matter lesions. Nguyen et al. (81) performed WBRT on 27 patients who failed HD-MTX treatment, and the results showed that the radiological ORR was 74%, the median survival time was 10.9 months, and 15% of patients had delayed neurotoxicity.

Stereotactic radiosurgery (SRS) is mainly used for the palliative treatment of patients with relapsed/refractory (R/R) PCNSL. Retrospective studies of small samples have suggested that SRS has a relatively higher local control rate for smaller tumors, but patients still have poor PFS and OS (83–85). Recently, the International Gamma Knife Research Foundation evaluated the role of SRS in the prognosis of relapsed PCNSL. Overall, 23 patients with R/R PCNSL were subjected to a total of 26 SRS programs, the median tumor volume was 4 cm^3^, and the median marginal dose was 15 Gy. Based on the results, 23 tumors of 20 patients had responses, and 14 patients had local control rates of 95%, 91% and 75% at 3, 6 and 12 months after SRS, respectively. The 1-year PFS rate and OS rate were 55% and 47%, respectively. SRS was also relatively less toxic, suggesting that SRS can be a part of multimodal management in patients with relapsed PCNSL (2).

### Hematopoietic Stem-Cell Transplantation

Patients who do not receive any consolidation therapy after HD-MTX rechallenge, are sensitive to second-line chemotherapy, or have not previously received HDC-ASCT should give priority to HDC-ASCT (60, 69); it also has similar promising outcomes for elderly patients in good general condition with relapse (86). The cohort study of PCNSL-relapsed patients by Soussain et al. (87) showed that the 5-year event-free survival (EFS) and OS rates of patients treated with thiotepa-based intensive chemotherapy plus hematopoietic stem cell rescue were 37.8% and 51.4%, respectively. Choi et al. (88) demonstrated that 18 patients who received ASCT had significantly better mPFS than patients who only received salvage chemotherapy (19.5 months vs 6.7 months). Multivariate analysis showed that no ASCT was associated with poor survival outcomes. Kasenda et al. (89) performed a prospective multicenter study of HDC-ASCT in 39 patients with R/R PCNSL who failed HD-MTX. Overall, 32 patients underwent HDC-ASCT, 56.4% had CR, the mPFS was 12.4 months, and the 2-year OS rate was 56.4%. A second relapse of PCNSL results in a poor prognosis; nevertheless, multiple long-term responses can be induced by repeated HD-MTX-based chemotherapy followed by HDC-ASCT in patients with eligible MTX-sensitive PCNSL (90). HDC-ASCT is also feasible and effective in elderly patients; a retrospective multicenter study by Schorb E et al. (86) showed that in elderly PCNSL patients (≥65 years) who received HDC-ASCT, 69.2% achieved CR, 17.3% achieved PR, and mPFS and OS were reached after 51.1 and 122.3 months, respectively, with a median follow-up of 22 months, though its efficacy still needs further support from prospective research.

### Targeted Therapy and Immunotherapy

**Figure 2:**
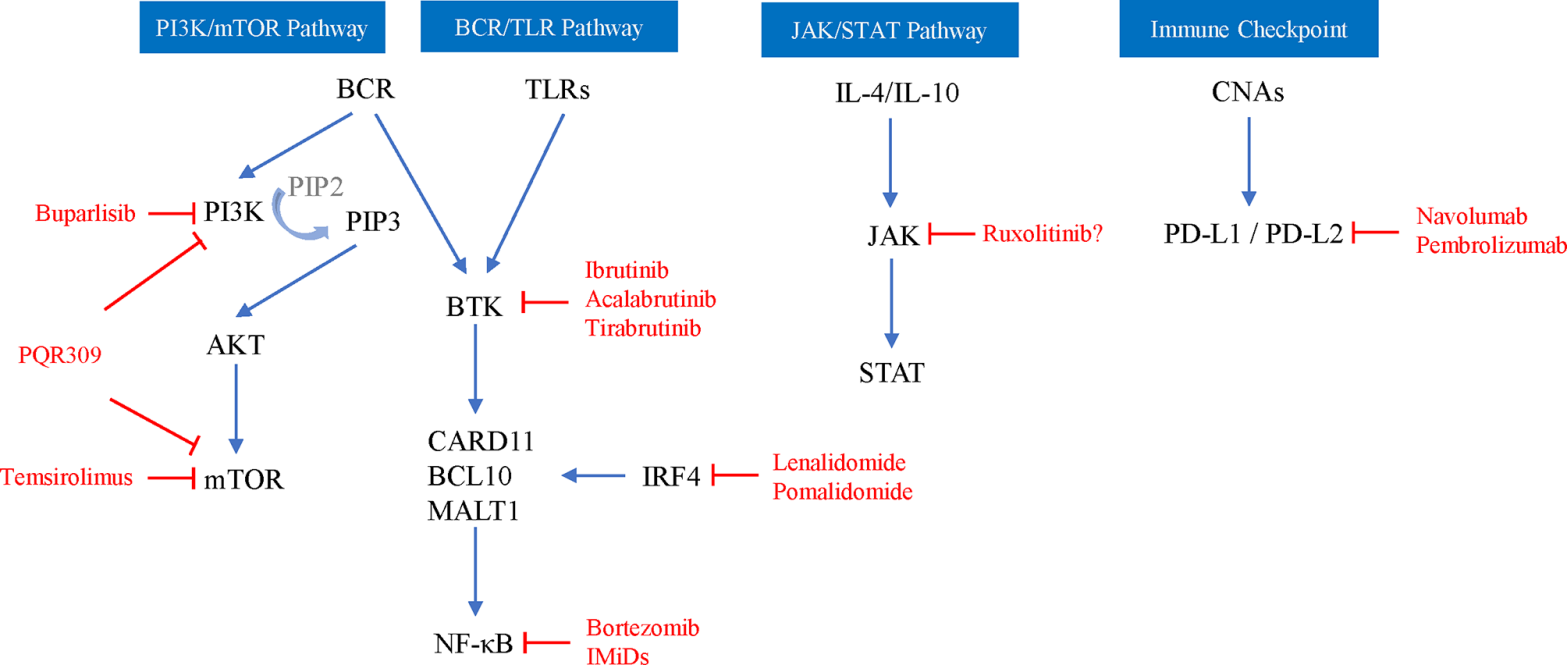
Schematic depiction of key mechanisms of PCNSL and related targeted agents (red words). PIP2, 3,4-diphosphate phosphatidylinositol; PIP3, 3,4,5-triphosphate phosphatidylinositol; AKT, serine/threonine kinase; CARD11, caspase recruitment domain-containing protein 11; BCL10, B-cell lymphoma 10 protein; MALT1, mucosa-associated lymphoid tissue lymphoma translocation protein 1; IRF4, interferon regulatory factor 4; CNAs, copy number alterations.

Limited studies have evaluated the ORR of monotherapy in recurrent/progressive PCNSL, showing 33-100% and an mOS of 4 months-981 days (**Table 1**), among which targeted therapy and immunotherapy are the most promising new strategies targeting the key mechanism of PCNSL (**Figure 2**) and show a higher ORR and fewer side effects than traditional cytotoxic drugs (5, 91–101).

**Table 1:** Selected trials on mono-chemotherapy for relapsed/refractory PCNSL.

	Study	Study design	Sample	Median age (years)	Mono-chemotherapy	Mechanism of chemotherapeutic	Administration	ORR (CR, PR) (%)	mPFS/mOS (months)	Grade 3/4/5 toxicity
Traditional Chemotherapeutics	Fischer et al. (91)	prospective	27	51	Topotecan	Topoisomerase I inhibitors	1.5mg/m^2^/d × 5days, every 3 weeks	33 (18, 15)	EFS 2.0/8.4	Leukopenia (26%) Neutropenia (11%) Thrombocytopenia (11%) Infection (11%) Anemia (3.7%) Non-hematologic toxicity (7%)
	Voloschin et al. (92)	prospective	15	56	Topotecan	Topoisomerase I inhibitors	1.5mg/m^2^/d × 5 days, every 3 weeks	40 (20, 20)	2/981days	Neutropenia (73%) Thrombocytopenia (20%)
	Raizer et al. (93)	NA	11	69.8	Pemetrexed	Multitarget antifolate	900 mg/m^2^/d, every 3 weeks	55 (36.3, 18.2)	5.7/10.1	Thrombocytopenia (45%) Leukopenia (36%) Anemia (27%) Infection (36%) Abnormal liver function (9%)
	Makino et al. (94)	retrospective	17	68	Temozolomide	Alkylating	150–200 mg/m^2^ × 5 days, every 4 weeks	47 (29, 18)	1.9/6.7	Neutropenia (6%) Thrombocytopenia (6%)
	Chamberlain et al. (95)	retrospective	12	61.5	Bendamustine	Bifunctional alkylating	100 mg/m^2^/d × 2 days, every 4 weeks	50 (25, 25)	3.5/5.5	Lymphopenia (33%) Anemia (17%), nausea (17%) Fatigue (8%), hyperglycemia (8%) Neutrophil reduction (8%)
Targeted Therapy	Batchelor et al. (96)	prospective	12	64	Rituximab	Anti-CD20 monoclonal antibody	375 mg/m^2^/W × 8 weeks	36 (27, 9)	57 days/20.9	Allergic reaction, fatigue Anxiety, back pain (6%)
	Houillier et al. (97)	retrospective	6	73.5	Lenalidomide	Immunomodulator	25 mg/d × 21 days, every 4weeks	50 (33, 17)	NA/4	None
	Korfel et al. (5)	prospective	37	70	Temsirolimus	mTOR inhibitor	25 or 75mg/w	54 (13.5, 32.4, CRu 8)	2.1/NA	Hyperglycemia (29.7%) Thrombocytopenia (21.6%) Infection (19%) Anemia (10.8%) Rash (8.1%)
	Chamoun et al. (98)	retrospective	14	68	Ibrutinib	BTK inhibitor	560mg/d	50 (42.8, 57.2)	6/NA	neutropenic fever (7%) diarrhea (7%) peritumoral hemorrhage (7%)
	Grommes et al. (99)	prospective	13	69	Ibrutinib	BTK inhibitor	560mg/d or 840mg/d	77 (38.5, 38.5)	4.6/15	Lymphopenia (20%) Neutropenia (15%) Hyperglycemia (15%) Thrombocytopenia (10%) Leukopenia (10%) Pulmonary infection (10%) Anemia (5%) Hypertriglyceridemia (5%)
	Soussain et al. (100)	prospective	52	67.5	Ibrutinib	BTK inhibitor	560mg/d	DC=62 (19, 33)	4.8/19.2	Infections and infestations (4%) Cardiac disorders (2%) General disorders and administration site conditions (2%) Blood and lymphatic system disorders (8%) Eye disorders (2%) Investigations (8%)
	Nayak et al. (101)	NA	4 relapsed/refractory PCNSL 1 CNS relapsed PTL	64	Nivolumab	Anti-PD1 monoclonal antibody	3 mg/kg, every 2 weeks	100 (80, 20)	14^+^/NA	None

PTL, primary testicular lymphoma; NA, not available; DC, disease control.

Rituximab can downregulate IL-10 and Bcl-2 by inactivating signal transducers and STAT3. The ORR of rituximab monotherapy for recurrent PCNSL is 36%, the mPFS is 57 days, and the mOS is 20.9 months (96). Because rituximab can make CD20B lymphoma cells sensitive to cytotoxic chemotherapy, it is often used in combination with other chemotherapeutic drugs, and the overall toxicity of the treatment is low (74), but the RTX molecule is too large to cross the complete BBB, so its efficacy is still controversial. The combination of RTX+HD-MTX may not be an effective strategy for recurrent PCNSL. Miyakita et al. (102) conducted a retrospective observational study of 19 relapsed patients with a total of 30 relapses, and the results indicated that 66.6% of recurrences achieved CR/complete response unconfirmed (CRu), 13.3% achieved PR in the HD-MTX+RTX group, which was comparable to the HD-MTX monotherapy group, and the median time to tumor progression was not significantly different between the two groups. Similarly, in a prospective multicenter study of rituximab combined with temozolomide in recurrent PCNSL, CR was observed in only 14% of evaluable patients with an mPFS of 7 weeks, and mOS was not reached. Although the treatment is well-tolerated, preliminary analysis shows that this regimen is ineffective (103).

Inhibitors targeting the PI3K/AKT/mTOR signaling pathway mainly include PI3K inhibitors, mTOR inhibitors and dual PI3K-mTOR inhibitors. The efficacy and safety of these inhibitors in relapsed PCNSL patients are not as expected based on clinical studies, and it may be difficult to achieve long-term disease control. Buparlisib is a pan-PI3K inhibitor, although in vitro experiments have shown that it has a good effect on lymphoma cells; for example, the response rate in monotherapy of R/R PCNSL was only 25% due to the limited blood-brain barrier penetration (104). Temsirolimus is an intravenous mTOR inhibitor that easily crosses the BBB. A phase II study of temsirolimus monotherapy in R/R PCNSL showed a good ORR of 54% (CR13.5%, PR 32.4%, and CRu 8%), but the PFS was only 2.1 months with a treatment-associated mortality rate of 13.5% (5). PQR309 can inhibit both pan-PI3K and mTOR and has good BBB permeability. Currently, the phase II trial of PQR309 in R/R PCNSL (NCT02669511) is still ongoing.

BCR pathway activation is still considered to be the key pathogenesis of PCNSL, and current drugs for recurrent PCNSL therapy involved in this pathway mainly involve Bruton tyrosine kinase inhibitors (BTKIs) and immunomodulatory drugs (IMiDs). While proteasome inhibitors that inhibit NF-κB activation, such as bortezomib, have good sensitivity in MTX-resistant lymphoma cell lines, their clinical efficacy is still controversial (105). Ibrutinib, a BTKI, has clinical activity in the brain, CSF, and intraocular septum and can be tolerated by patients with R/R PCNSL. The ORR of R/R PCNSL patients treated with ibrutinib monotherapy is 50-77%, the mPFS is 4.6-6 months, and the mOS is 15-19.2 months (98–100). Although single-agent activity is shown in R/R PCNSL, the clinical response of patients is usually short-lived or incomplete, and combination therapy may be required. A phase 1b clinical trial explored the efficacy of ibrutinib/HD-MTX/rituximab combination therapy for R/R PCNSL, and 80% of patients had clinical responses without grade 3-5 toxicity events or dose-limiting toxicity (106). Lionakis et al. (107) performed ibrutinib combined with anthracycline-based chemotherapy (DA-TEDDi-R) on 18 PCNSL patients (including 13 R/R PCNSL); 86% of the assessed patients achieved CR and the mPFS was 15.3 months. In addition, acalabrutinib does a better job in attacking this target than ibrutinib, and a phase 2 trial of acalabrutinib in R/R PCNSL (NCT04548648) is currently ongoing. Ibrutinib inhibits a variety of off-target kinases and can cause serious toxicity. The second-generation highly selective oral BKTI tirabrutinib has shown good safety and efficacy in R/R PCNSL. The ORR with tirabrutinib was 64%; the mPFS was 2.9 months; the CR/CRu was 60%, 100% and 53%; and the mPFS was 2.1, 11.1 and 5.8 months under 320 mg, 480 mg and 480 mg fasting conditions, respectively (3).

IMiDs include lenalidomide and pomalidomide (POM), which not only inhibit the activity of NF-κB but also inhibit the PI3K/AKT pathway. Both have been shown to cross the BBB, and a preclinical model system shows that pomalidomide has a higher CNS permeability than lenalidomide (108, 109). Lenalidomide is a second-generation IMiD that is active as a single-agent maintenance therapy in relapsing PCNSL and significantly prolongs the duration of response (97). The study of Rubenstein et al. (110) showed that of 9 patients after low-dose lenalidomide maintenance treatment, 6 patients had maintained a response ≥ 9 months, 4 patients had a response ≥18 months, and the mOS was 45 months. The lenalidomide + rituximab (R2) regimen showed significant activity in R/R DLBCL-PCNSL patients, with an ORR of 35.6% and a mPFS and overall OS of 7.8 months and 17.7 months, respectively, without unexpected toxicity (111). Pomalidomide is a third-generation IMiD, and its combination with dexamethasone can significantly improve survival compared with IMiDs alone. Pomalidomide has specific activity in patients with PCNSL recurrence. Tun et al. (1) conducted a phase 1 study of 25 patients with PCNSL and PVRL treated with combined dexamethasone and POM, and the results showed that the ORR was 48% (6 CR, 2 CRu, and 4 PR), the mPFS was 5.3 months, the maximum tolerated dose cohort ORR was 50%, and the mPFS was 9 months.

After lenalidomide, ibrutinib, and rituximab, other new drugs have entered clinical trials one after another. For older PCNSL patients who relapse in extranodal areas (including the CNS), immune checkpoint inhibitors may be another promising method. Nivolumab is a human immunoglobulin G4 monoclonal antibody that targets PD-1 and blocks the binding of PD-1 ligands. In a retrospective study of 5 cases with R/R PCNSL and primary testicular lymphoma, all patients treated with navolumab achieved clinical and imaging responses, and 3 patients remained progression-free for 13-17 months (101). Terziev et al. (90) reported a case of PCNSL patients who achieved CR after a second relapse with nivolumab as maintenance therapy. The patient remained in remission for > 2 years and was in a good clinical state. The multicenter phase 2 clinical trial of nivolumab for R/R PCNSL has recently been finished, and the results showed that the ORR was 6.4%, the median PFS was 1.41 months, and the median OS was 8.64 months (NCT02857426). Another phase 2 clinical trial on pembrolizumab in R/R PCNSL is ongoing (NCT03255018). PD-1 inhibitors and rituximab combined immunotherapy may result in the activation of immune systems and in enhancing clinical efficacy. Ambady et al. (112) performed pembrolizumab/nivolumab+rituximab treatment on 3 patients with progressive PCNSL, and the total ORR was 2/3 (2 CR and 1 progression) and the duration of response 6-7 months by the end of follow-up.

Chimeric antigen receptor (CAR) T-cells targeting CD19 have been approved for the treatment of relapsed/refractory systemic diffuse large B-cell lymphoma, which can induce a DLBCL patient response at the rate of 64-86% (113). However, trials are needed to certify the efficacy and safety of CART in relapsed PCNSL; a phase 1 clinical trial on CAR T-cells in relapsed/refractory CD19^+^ PCNSL just started (NCT04443829).

Histone deacetylase inhibitors (HDACIs) have shown good efficacy in various types of intracranial metastatic tumors. In cases of refractory peripheral T-cell lymphoma with CNS metastasis, romidepsin achieves durable clinical remission (114). Patients with brain metastases from non-small cell lung cancer were treated with chidamide combined with paclitaxel and carboplatin, and 40% of patients achieved complete intracranial response after treatment (115). In vitro experiments have proven that HDACIs can enhance the therapeutic effect of MTX by increasing the polyglutamylation of MTX and downregulating DHFR expression (116). HDACIs are expected to be quickly evaluated in recurrent PCNSL.

### Treatment of Special Relapses

There is substantial heterogeneity in salvage therapy for patients with isolated systemic relapses and relapses such as NL and lymphocytic meningitis, and related studies are lacking. Patients with isolated systemic relapses usually receive chemotherapy alone (e.g., CHOP, R-CHOP, ACVBP, DHAP, and R-hyper CVAD), surgery alone, radiation therapy alone, hematopoietic stem cell transplantation or combination. The CR rate is 50-83%, and the mOS after relapse is 15.5 months (19, 35, 45, 47). Most relapsed patients with NL receive chemotherapies based on high-dose MTX or cytarabine and radiation therapy, while patients with brain/spinal root involvement or higher CSF cell counts receive intrathecal chemotherapy, and the survival rates at 12 months and 36 months are approximately 46% and 24%, respectively (50, 51). In a study of lymphocytic meningitis by Chamberlain et al. (53), 2 patients underwent WBRT, 8 patients received systemic chemotherapy (e.g., high-dose MTX or cytarabine), and 7 patients received intraventricular chemotherapy without high-dose systemic chemotherapy; the ORR was 28.6% and the median survival was 5.5 months.

## Prognosis

The prognosis of patients with relapsed PCNSL is very poor, the median survival time without additional treatment is only 2 months, the median survival time from the first disease progression to death from any cause is 7.2 months, and the survival time is within 2 years (42, 66, 70, 82, 111). There are many factors affecting prognosis, including age, treatment sensitivity, salvage therapeutic schemes, relapse location, relapse time, etc., but in general, salvage treatment and patient recurrence time are still important factors affecting the prognosis and quality of life (4, 6, 17, 19, 35, 42, 117).

## Future Directions

In the past 20 years, the oncology community has made significant progress in the understanding of the pathogenesis and treatment of PCNSL. There are more and more treatment options for patients with relapsed PCNSL, and some drugs have been included in the NCCN guidelines for the treatment of R/R PCNSL. However, there are still many challenges in the follow-up and treatment of relapsed PCNSL, especially the limited diagnosis rate by traditional imaging in follow-up and the personalized selection of therapeutic schemes, though some novel technologies may provide us additional help. Machine learning based on MRI has shown good results in the application of distinguishing PCNSL from other CNS tumors (118, 119). In the future, machine learning based on imaging of PCNSL recurrence may help the early and accurate diagnosis compared with manual reading. The development of next-generation sequencing has allowed individuals to identify more tumor-driving mutations or specific molecular signatures related to the pathogenesis and prognosis of PCNSL (28, 120, 121) and to determine more targeted pathways of this disease for the development of more small molecule drugs with good blood-brain barrier permeability. For patients with relapsed PCNSL, individualized treatments based on genetic screening can also be performed to improve prognosis. In addition, the current common treatment options for relapse still lack the support of large-scale prospective trials, and some small molecule drugs have a limited response duration. Therefore, more prospective studies, especially on reasonable combination strategies of small molecule drugs, are needed in the future.

## Conclusions

The relapse rate of primary central nervous system lymphoma is high, there are many related factors for recurrence, and the clinical manifestations are diverse. Based on the understanding of the pathophysiology of PCNSL, an increasing number of targeted drugs and immunotherapies have been introduced to salvage clinical trials and have shown good clinical response, but the prognosis of relapsed patients is still poor. There is still a lack of prospective multicenter studies to seek better treatment options.

## Author Contributions

KT: Development of all topics and table creation, writing, and revision of the entire article. XW and XT: General idea and advice of the whole article. All authors contributed to the article and approved the submitted version.

## Funding

This work was funded by the National Natural Science Foundation of China (grant number 82001378 to XT).

## Conflict of Interest

The authors declare that the research was conducted in the absence of any commercial or financial relationships that could be construed as a potential conflict of interest.
